# Supplementary material for: EnzML: multi-label prediction of enzyme classes using InterPro signatures
Source: BMC Bioinformatics. 2012 Apr 25;13:61. doi: 10.1186/1471-2105-13-61 (PMC3483700; doi:10.1186/1471-2105-13-61)
Supplement: Addtional file 5 — The Java code to format the data files, evaluate and predict. The file enzml_java_code.tar.gz contains the Java code used to format database data to ARFF and XML formats, to execute cross and train-test (jackknife) evaluations and to record evaluation results to database. More information is included in the readme.txt file and the Javadoc files. The code can be used with a MySQL database. To use a different database software, other JDBC drivers might be required. [file 1471-2105-13-61-S5.gz › java_code/enzml2011/doc/uk/ac/ed/inf/enzml/test/mulan/predict/MulanPredictTest.html]

MulanPredictTest


---


|  |  |  |
| --- | --- | --- |
| **Overview** \| **Package** \| **Class** \| **Use** \| **Tree** \| **Deprecated** \| **Index** \| **Help** | |  |
| **PREV CLASS**   NEXT CLASS | **FRAMES**    **NO FRAMES**     **All Classes** |  |
| SUMMARY: NESTED \| FIELD \| CONSTR \| METHOD | DETAIL: FIELD \| CONSTR \| METHOD |  |


---


## uk.ac.ed.inf.enzml.test.mulan.predict Class MulanPredictTest

```
java.lang.Object
  junit.framework.Assert
      junit.framework.TestCase
          uk.ac.ed.inf.enzml.test.mulan.predict.MulanPredictTest
```

**All Implemented Interfaces:**: junit.framework.Test

---

``` public class MulanPredictTest extends junit.framework.TestCase ```

Train arff: inst1, inst2 etc. att 1, 3, 4, 5 class 1.1.1.1, 2.2.2.2
Test arff: instA, instB, instC etc. att 2, 3 class 2.2.2.2, 3.3.3.3
Test data filtered by train: cannot contain att 2, must contain att1, 3, 4, 5
Types comment: change at eclipse > java > code style > code template > Types

---

**Constructor Summary**

`MulanPredictTest()`


| **Method Summary** | |
| --- | --- |
| `static MulanPredict` | `getMulanPredict(int trainId, int testId)` |
| `static MulanPredict` | `getMulanPredictAttributesFiltered()` |
| `static MulanPredict` | `getMulanPredictPlain()` |
| `void` | `testHeader(java.lang.String predictions)` |
| `void` | `testPrintPredictions()` |
| `void` | `testPrintPredictionsAttributesFiltered()` |

**Methods inherited from class junit.framework.TestCase**

`countTestCases, getName, run, run, runBare, setName, toString`

**Methods inherited from class junit.framework.Assert**

`assertEquals, assertEquals, assertEquals, assertEquals, assertEquals, assertEquals, assertEquals, assertEquals, assertEquals, assertEquals, assertEquals, assertEquals, assertEquals, assertEquals, assertEquals, assertEquals, assertEquals, assertEquals, assertEquals, assertEquals, assertFalse, assertFalse, assertNotNull, assertNotNull, assertNotSame, assertNotSame, assertNull, assertNull, assertSame, assertSame, assertTrue, assertTrue, fail, fail, failNotEquals, failNotSame, failSame, format`

**Methods inherited from class java.lang.Object**

`equals, getClass, hashCode, notify, notifyAll, wait, wait, wait`

**Constructor Detail**

### MulanPredictTest

```
public MulanPredictTest()
```


**Method Detail**

### testHeader

```
public void testHeader(java.lang.String predictions)
```

---


### testPrintPredictions

```
public void testPrintPredictions()
```

---


### testPrintPredictionsAttributesFiltered

```
public void testPrintPredictionsAttributesFiltered()
```

---


### getMulanPredict

```
public static MulanPredict getMulanPredict(int trainId,
                                           int testId)
```

---


### getMulanPredictAttributesFiltered

```
public static MulanPredict getMulanPredictAttributesFiltered()
```

---


### getMulanPredictPlain

```
public static MulanPredict getMulanPredictPlain()
```


---


|  |  |  |
| --- | --- | --- |
| **Overview** \| **Package** \| **Class** \| **Use** \| **Tree** \| **Deprecated** \| **Index** \| **Help** | |  |
| **PREV CLASS**   NEXT CLASS | **FRAMES**    **NO FRAMES**     **All Classes** |  |
| SUMMARY: NESTED \| FIELD \| CONSTR \| METHOD | DETAIL: FIELD \| CONSTR \| METHOD |  |


---
